# Pleomorphic xanthoastrocytoma is a heterogeneous entity with *pTERT* mutations prognosticating shorter survival

**DOI:** 10.1186/s40478-021-01308-1

**Published:** 2022-01-10

**Authors:** Azadeh Ebrahimi, Andrey Korshunov, Guido Reifenberger, David Capper, Joerg Felsberg, Elena Trisolini, Bianca Pollo, Chiara Calatozzolo, Marco Prinz, Ori Staszewski, Leonille Schweizer, Jens Schittenhelm, Patrick N. Harter, Werner Paulus, Christian Thomas, Patricia Kohlhof-Meinecke, Marcel Seiz-Rosenhagen, Till Milde, Belén M. Casalini, Abigail Suwala, Annika K. Wefers, Annekathrin Reinhardt, Philipp Sievers, Christof M. Kramm, Nima Etminam, Andreas Unterberg, Wolfgang Wick, Christel Herold-Mende, Dominik Sturm, Stefan M. Pfister, Martin Sill, David T. W. Jones, Daniel Schrimpf, David E. Reuss, Ken Aldape, Zied Abdullaev, Felix Sahm, Andreas von Deimling, Damian Stichel

**Affiliations:** 1 Department of Neuropathology, Institute of Pathology, University of Heidelberg, Im Neuenheimer Feld 224, 69120 Heidelberg, Germany; 2 Clinical Cooperation Unit Neuropathology, German Cancer Consortium (DKTK), German Cancer Research Center (DKFZ), Heidelberg, Germany; 3 Institute of Neuropathology, Heinrich Heine University, Duesseldorf, Germany; 4 German Cancer Consortium (DKTK), Partner Site, Essen/Duesseldorf, Germany; 5 German Cancer Consortium (DKTK), Partner Site Berlin, German Cancer Research Center (DKFZ), Heidelberg, Germany; 6 Department of Neuropathology, Charité-Universitaetsmedizin Berlin, Corporate Member of Freie Universitaet Berlin, Humboldt-Universitaet Zu Berlin, and Berlin Institute of Health, Berlin, Germany; 7 Pathology Unit, Maggiore Della Carità Hospital, 28100 Novara, Italy; 8 Neuropathology Unit, Fondazione IRCCS Istituto Neurologico Carlo Besta, Milan, Italy; 9 Institute of Neuropathology, Faculty of Medicine, University of Freiburg, Freiburg, Germany; 10 Signalling Research Centres BIOSS and CIBSS, University of Freiburg, Freiburg, Germany; 11 Center for Basics in NeuroModulation (NeuroModulBasics), Faculty of Medicine, University of Freiburg, Freiburg, Germany; 12 Department of Neuropathology, Institute of Pathology and Neuropathology, University Hospital of Tuebingen, Eberhard Karls University of Tuebingen, 72076 Tuebingen, Germany; 13 Center for CNS Tumors, Comprehensive Cancer Center Tuebingen-Stuttgart, University Hospital of Tuebingen, Eberhard Karls University of Tuebingen, Tuebingen, Germany; 14 Institute of Neurology (Edinger Institute), Goethe University, Frankfurt, Germany; 15 German Cancer Consortium (DKTK), Partner Site Frankfurt/Mainz, Frankfurt am Main, Germany; 16 German Cancer Research Center (DKFZ), Heidelberg, Germany; 17 Frankfurt Cancer Institute (FCI), Frankfurt am Main, Germany; 18 Institute of Neuropathology, University Hospital Muenster, Pottkamp 2, Muenster, Germany; 19 Department of Pathology, Katharinenhospital Stuttgart, Stuttgart, Germany; 20 Department of Neurosurgery, Hospital Memmingen, Memmingen, Germany; 21 Division of Pediatric Neurooncology, German Cancer Consortium (DKTK) and German Cancer Research Center (DKFZ), 69120 Heidelberg, Germany; 22 Division of Pediatric Hematology and Oncology, University Medical Center Göttingen, Göttingen, Germany; 23 Department of Neurosurgery, Medical Faculty Mannheim, University Hospital Mannheim, University of Heidelberg, Theodor-Kutzer-Ufer 1-3, 68167 Mannheim, Germany; 24 Division of Experimental Neurosurgery, Department of Neurosurgery, Heidelberg University Hospital, Heidelberg, Germany; 25 Department of Neurology and Neurooncology Program, National Center for Tumor Diseases, Heidelberg University Hospital, Heidelberg, Germany; 26 Clinical Cooperation Unit Neurooncology, German Cancer Consortium (DKTK), German Cancer Research Center (DKFZ), Heidelberg, Germany; 27 Hopp Children’s Cancer Center Heidelberg (KiTZ), Heidelberg, Germany; 28 Department of Pediatric Oncology, Hematology and Immunology, Heidelberg University Hospital, Heidelberg, Germany; 29 Pediatric Glioma Research Group, German Cancer Research Center (DKFZ), 69120 Heidelberg, Germany; 30 Laboratory of Pathology, National Cancer Institute, Centre for Cancer Research, Bethesda, MD USA; 31 Clinical Cooperation Unit Pediatric Oncology, German Cancer Research Center (DKFZ) and German Consortium for Translational Cancer Research (DKTK), Heidelberg, Germany; 32 Institute of Neuropathology, University of Bonn, Venusberg-Campus 1, 53127 Bonn, Germany

**Keywords:** Pleomorphic xanthoastrocytoma, Ganglioglioma, Epithelioid glioblastoma, *BRAF V600E*, *pTERT* mutation, DNA methylation array profiling

## Abstract

Pleomorphic xanthoastrocytoma (PXA) in its classic manifestation exhibits distinct morphological features and is assigned to CNS WHO grade 2 or grade 3. Distinction from glioblastoma variants and lower grade glial and glioneuronal tumors is a common diagnostic challenge. We compared a morphologically defined set of PXA (histPXA) with an independent set, defined by DNA methylation analysis (mcPXA). HistPXA encompassed 144 tumors all subjected to DNA methylation array analysis. Sixty-two histPXA matched to the methylation class mcPXA. These were combined with the cases that showed the mcPXA signature but had received a histopathological diagnosis other than PXA. This cohort constituted a set of 220 mcPXA. Molecular and clinical parameters were analyzed in these groups. Morphological parameters were analyzed in a subset of tumors with FFPE tissue available. HistPXA revealed considerable heterogeneity in regard to methylation classes, with methylation classes glioblastoma and ganglioglioma being the most frequent mismatches. Similarly, the mcPXA cohort contained tumors of diverse histological diagnoses, with glioblastoma constituting the most frequent mismatch. Subsequent analyses demonstrated the presence of canonical *pTERT* mutations to be associated with unfavorable prognosis among mcPXA. Based on these data, we consider the tumor type PXA to be histologically more varied than previously assumed. Histological approach to diagnosis will predominantly identify cases with the established archetypical morphology. DNA methylation analysis includes additional tumors in the tumor class PXA that share similar DNA methylation profile but lack the typical morphology of a PXA. DNA methylation analysis also assist in separating other tumor types with morphologic overlap to PXA. Our data suggest the presence of canonical *pTERT* mutations as a robust indicator for poor prognosis in methylation class PXA.

**Supplementary Information:**

The online version contains supplementary material available at 10.1186/s40478-021-01308-1.

## Introduction

Pleomorphic xanthoastrocytoma (PXA) was recognized as a distinct entity in 1979 [10]. PXA may present with or without morphological signs of malignancy. On the molecular level, homozygous deletion of *CDKN2A/B* (60–95%) and *BRAF V600E* mutation (60–78%) have emerged as predominant features, each seen in the majority of PXA [4, 8, 12, 20, 21, 29]. Furthermore, PXA exhibits a characteristic DNA methylation profile allowing clear separation from other tumor entities such as ganglioglioma and glioblastoma [2].

There appears to be a morphological continuum between anaplastic PXA and glioblastoma. Epithelioid glioblastoma, a recently described histologic variant [14], shares both morphological as well as molecular features with anaplastic PXA [11], including a high incidence of *BRAF V600E* mutation. On the benign end of the malignancy spectrum, PXA can also harbor morphologic and molecular similarities to lower grade tumors such as ganglioglioma. Gangliogliomas also carry *BRAF V600E* mutation in the majority of the cases and have been reported occasionally to contain *CDKN2A/B* homozygous deletions [19]. This renders PXA difficult to distinguish from morphological mimics on both ends of the malignancy spectrum.

Developing criteria for grading of PXA has been a challenge. Malignant progression of PXA has been repeatedly reported [17, 18, 24]. The count of ≥ 5 mitoses per 10 high-power fields (corresponding to 4 mm^2^) has been introduced as an indicator of poor prognosis [8] and this was included in the 2016 and 2021 WHO classification of tumors of central nervous system as an important criterion for CNS WHO grade 3 (former WHO grade III) [14, 15]. Few reports have *introduced BRAF V600E* mutation as a molecular prognosticator, albeit with conflicting interpretations [8, 20, 23, 26].

In the present study, a multicenter cohort of PXA based on histological diagnosis (histPXA) was subjected to DNA methylation-based classification, in order to obtain information on the heterogeneity within this set. A second cohort (mcPXA) was established by mining a large repository for tumors exhibiting a methylation profile assigned to PXA.

## Material and methods

### Patient cohorts

HistPXA were collected from the archives of the Departments of Neuropathology, Heidelberg, Mannheim, Duesseldorf, Frankfurt, Berlin, Tuebingen, Freiburg, Muenster, Stuttgart, Milan, Novara and Moscow. All 144 HistPXA cases had received a neuropathological evaluation in the original institute. These cases were subjected to DNA methylation array analysis (Additional file 1: Table 1). In a reverse approach, we identified 220 tumors in our data bank with the signature of methylation class PXA (mcPXA) and a calibrated score of 0.9 or higher, irrespective of initial histological diagnosis (Additional file 2: Table 2). McPXA also included 62 cases of the histPXA group exhibiting a calibrated score of 0.9 or higher. Survival data were collected if possible. Among those cases with available survival (77), 46 cases with sufficient material were subjected to additional morphological evaluation in Heidelberg. In addition, 1105 tumors with the DNA methylation signature glioblastoma, *IDH*-wildtype (mcGBM including subgroups GBM RTK I, GBM RTK II, GBM MES) were selected to serve as control cohort for survival and copy number alterations. Information regarding the composition of cohorts and sample sizes are also available in Additional file 3.

### Histology and immunohistochemistry

All cases were diagnosed at the local neuropathology centers. Given the availability of material, additional histopathological review on H&E- and Ki67-staining of samples was done by two experienced neuropathologists in Heidelberg. Histopathological evaluation was based on the 2016 WHO classification of tumors of central nervous system and later adapted to the newest guidelines of 2021 WHO [14, 15]. These criteria has not changed according to the newest guidelines of 2021 WHO except the Roman numbering system that has been changed to the Arabic numbering system [15]. Immunohistochemical staining for Ki67 was performed on a Ventana BenchMark Ultra Immunostainer using the OptiView DAB IHC Detection Kit. The Mouse monoclonal antibody, Clone MIB-1, Dako was used with a dilution of 1:100, pretreatment CC1 according to OptiView; Agilent protocol. Stained slides were scanned on an Aperio AT2 Scanner (Aperio Technologies, Vista, California, USA) and photographed using Aperio ImageScope software v12.3.2.8013.

### DNA extraction

DNA was extracted from FFPE material. Using H&E staining, areas with the highest tumor cell content were selected and DNA was extracted automatically, using a Maxwell system (Promega, Fitchburg, WI, USA) and the Maxwell 16 FFPE Plus LEV DNA Purification Kit according to the manufacturer’s instructions. The DNA concentrations were determined with the Invitrogen Qubit dsDNA BR Assay Kit (Thermo Fisher Scientific, Waltham, MA, USA), and a FLUOstar Omega Microplate Reader (BMG Labtech GmbH, Ortenberg, Germany).

### DNA methylation profiling

In order to obtain genome-wide DNA methylation profiles of tumor samples, the Infinium HumanMethylation450 (450 k) BeadChip or Infinium Methylation EPIC (850 k) BeadChip array (Illumina, San Diego, CA, USA) was used according to the manufacturer’s instructions at the Genomics and Proteomics Core Facility of the German Cancer Research Center (DKFZ; Heidelberg, Germany) [2]. All samples were carefully controlled for their on-chip quality metrics. Processing of DNA methylation data was performed with custom approaches, as previously described [2]. The copy number variation plots were generated from the raw data using the ‘conumee’ R package in Bioconductor (http://www.bioconductor.org.packages/release/bioc/html/conumee.html). Automated assessment of copy-number changes was performed using the results from conumee after additional baseline correction. Sample duplicates were ruled out by pairwise correlation of the genotyping probes on the 450 k/850 k array. Brain tumor classifier version v11b4 was applied for classification of tumors and all cases with a calibrated score of 0.9 or higher were considered as classifiable [2]. Those with a calibrated score less than 0.9 received a group assignment based on tSNE analysis [25] with a set of reference samples underlying the classifier version v11b4 (Additional file 1: Table 1) [2]. The tSNE was computed using the R-package Rtsne (https://github.com/jkrijthe/Rtsne) and the 10,000 most variable methylated CpG sites according to the standard deviation, 3000 iterations and a perplexity value of 10.

Numerical alterations of the genes with established relevance for astrocytic gliomas, comprising *CCND1, CCND2, CDK4, CDK6, CDKN2A/B, EGFR, MDM4, MET, MYC, MYCN, NF1, NF2, PDGFRA, PPM1D, PTEN* and *RB1* were evaluated as described [22]. Tumor purity was calculated using the tool RF_Purify [9]. Targeted Sanger sequencing of *BRAF* and *pTERT* was performed on PXAs providing sufficient DNA availability.

#### Statistics

Kaplan–Meier analysis was used for survival analysis, with a log-rank test performed for comparing groups. Fisher's exact test was used for comparing independent groups. Software R version 3.4 as well as statistical software SAS JMP version 15 were employed for analysis. P value < 0.05 was considered as significant.

## Results

### HistPXA encompasses a diverse spectrum of molecular classes

**Fig. 1:**
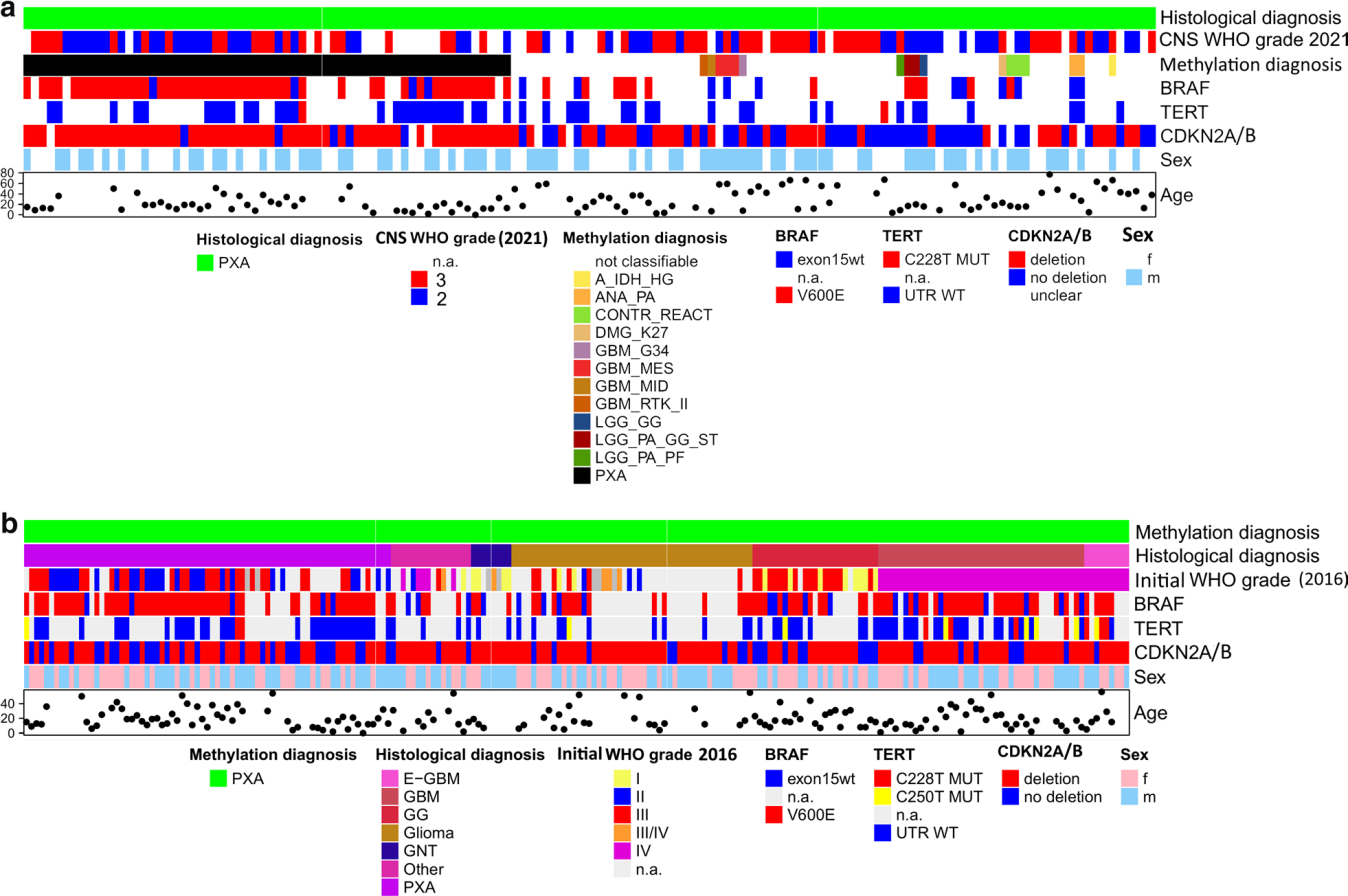
(**a**) Overview of cases with initial histological diagnosis PXA (histPXA) and assignment to classes via the brain tumor classifier. For more information about methylation classes please refer to our previous publication [2] (**b**) Spectrum of histological diagnoses of cases assigned to methylation class PXA (mcPXA; GBM = glioblastoma and gliosarcoma E-GBM = epithelioid glioblastoma, Glioma = other glial tumors including diffuse astrocytoma, oligodendroglioma, ependymoma, pilocytic astrocytoma, astroblastoma and glioma without further specification, GG = ganglioglioma and GNT = other glioneuronal tumors, PXA = pleomorphic xanthoastrocytoma and anaplastic pleomorphic xanthoastrocytoma; other = included further rare diagnoses like PNET, meningioma, ATRT, FCD, etc. A comprehensive list of abbreviations is provided in the Additional file 6

Among 144 histPXA, 79 had a maximum calibrated score of 0.9 or higher and were assigned to distinct methylation classes by the brain tumor classifier (version v11b4) including 62 mcPXA (Fig. 1a). 65 tumors had a maximum calibrated score lower than 0.9. These were subjected to tSNE-analysis (Additional file 4). From those, 48 cases were attributed to a methylation class (Fig. 1a, Additional file 1: Table 1). Seventeen cases could not be clearly assigned to any established group. The average purity (mean RF_ABSOLUTE Purity score) in non-classifiable cases was 0.5 similar to the value of 0.5 for mcPXA cases. Thus, there was no correlation between DNA purity and class prediction. GBM was the second most common methylation class among histPXA (n = 6 via v11b4, n = 14 via tSNE) followed by ganglioglioma (n = 3 via v11b4, n = 6 via tSNE,)

### WHO grading separates histPXA into groups with different clinical outcome

**Fig. 2:**
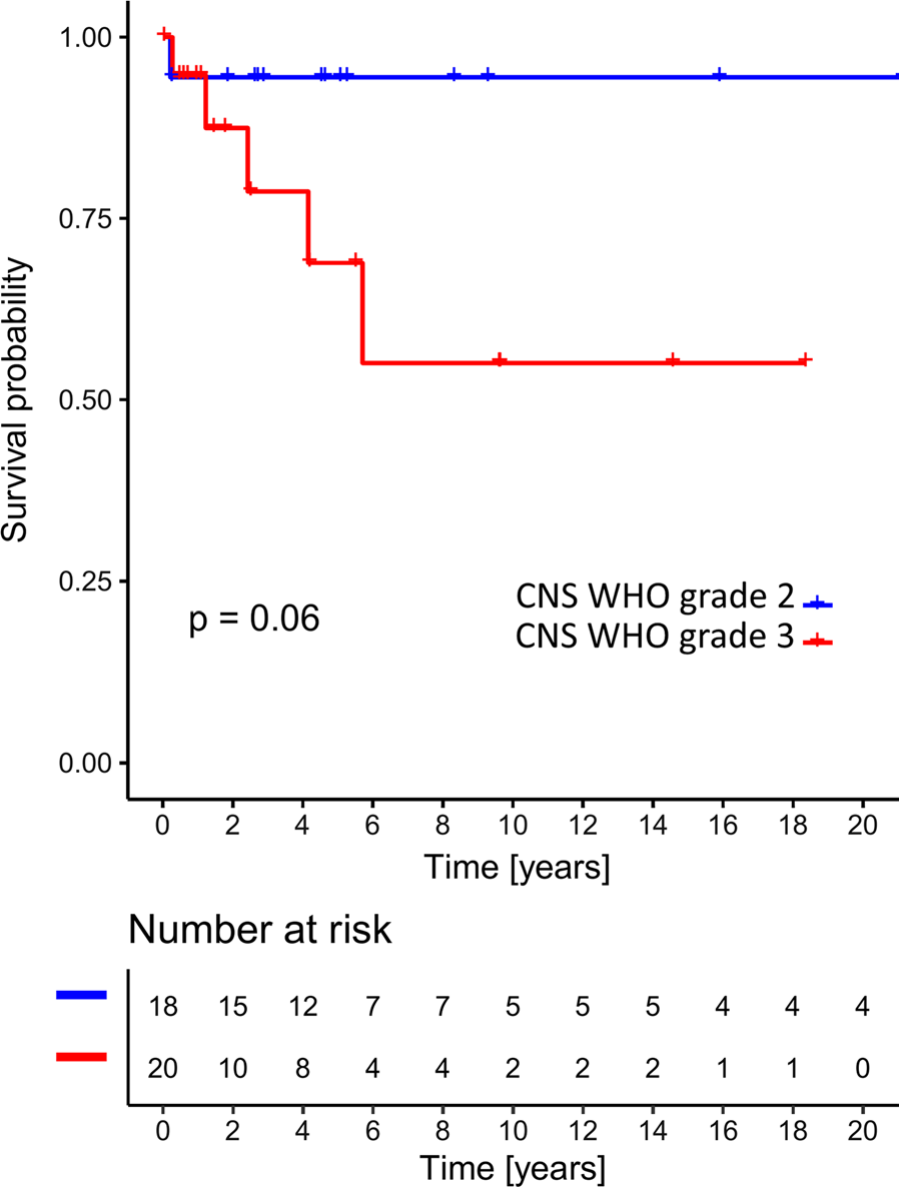
Overall survival (Kaplan–Meier curve) of histPXA CNS WHO grade 2 and 3 without accounting for methylation class n = 38, log rank p = 0.06

Kaplan–Meier survival analysis of 38 histPXA without accounting for underlying methylation groups highlighted the differences in survival curves of CNS WHO grade 2 and 3 Tumors (Fig. 2). However, when cases with both histPXA diagnosis and mcPXA signature according to v11b4 (n = 17) were analyzed by Kaplan–Meier analysis, CNS WHO grade was not associated with different outcome (p = 0.48). In this case, the low number of cases with outcome data may compromise this distribution. Adding other unclassifiable PXA cases recruited by tSNE evaluation to the mcPXA cluster (n = 21), did not lead to significant results.

### McPXA includes a diverse spectrum of histological diagnoses

McPXA comprised all tumors with the signature of methylation class PXA (n = 220). The most frequent histological diagnosis was PXA (n = 73). The remaining 147 cases exhibited a spectrum of histological diagnoses, mostly low- and high-grade gliomas (n = 99). Among these, glioblastoma (n = 50) including nine epithelioid glioblastoma was the most common diagnosis followed by other gliomas (n = 50), ganglioglioma with other neuronal/glioneuronal tumors (n = 31) and “other” diagnoses (together n = 16; Fig. 1b, Additional file 2: Table 2).

### Morphology of mcPXA

FFPE material from 46 mcPXA with a calibrated score more than 0.9 and with available survival data was used for a neuropathological reevaluation in Heidelberg (Additional file 2: Table 2). There was a concordance of about 95% between the two neuropathologists. In non-concordant cases (n = 2) there was a slight discrepancy of mitotic count per 10 HPF between the two neuropathologists that did not affect the WHO grading of the tumor and in both cases the higher mitotic count was chosen. These 46 tumors were initially diagnosed as PXA (n = 20), glioblastoma including one epithelioid glioblastoma (n = 10), ganglioglioma (n = 8), other gliomas including pilocytic astrocytoma, anaplastic pilocytic astrocytoma, anaplastic diffuse astrocytoma, low grade glioma, pleomorphic glioma and astroblastoma and one tumor with unspecific histological diagnosis (altogether n = 7).

Applying WHO criteria for PXA grading to this set of mcPXA resulted in 19 “grade 2” (M < 5/10 HPF) and 27 “grade 3” (M ≥ 5/10 HPF) mcPXAs. Ki-67 proliferation index was higher (p = 0.0002) in “WHO grade 3” (17 ± 2) than in “grade 2” (7 ± 2). Necrosis was a common finding. Forty-four percent (20 of 46) exhibited necrosis from which approximately half (11 of 20) were of the pseudopalisading type (Additional file 5: Fig. 1e). Necrosis was more common in “grade 3” (n = 17) than in “grade 2” tumors (n = 3; p = 0.002). With the exception of one case, pseudopalisading necrosis was only observed in “grade 3” tumors. Endothelial proliferation (37 of 46) and thrombosed vessels (22 of 46) were common histological features. Thrombosed vessels were observed in both “grade 2” (6 of 19) and “grade 3” (16 of 27) tumors. Foci of calcification could be found in nearly one third of the cases (13 of 46). Three CNS WHO grade 3 tumors had small-round-blue-cell morphology. Additional histological features were eosinophilic granular bodies, perivascular inflammatory infiltrates, extensive myxoid matrix, and areas of previous bleeding (Additional file 5: Fig. 1).

### CNS WHO grading parameters do not stratify mcPXA for survival

**Fig. 3:**
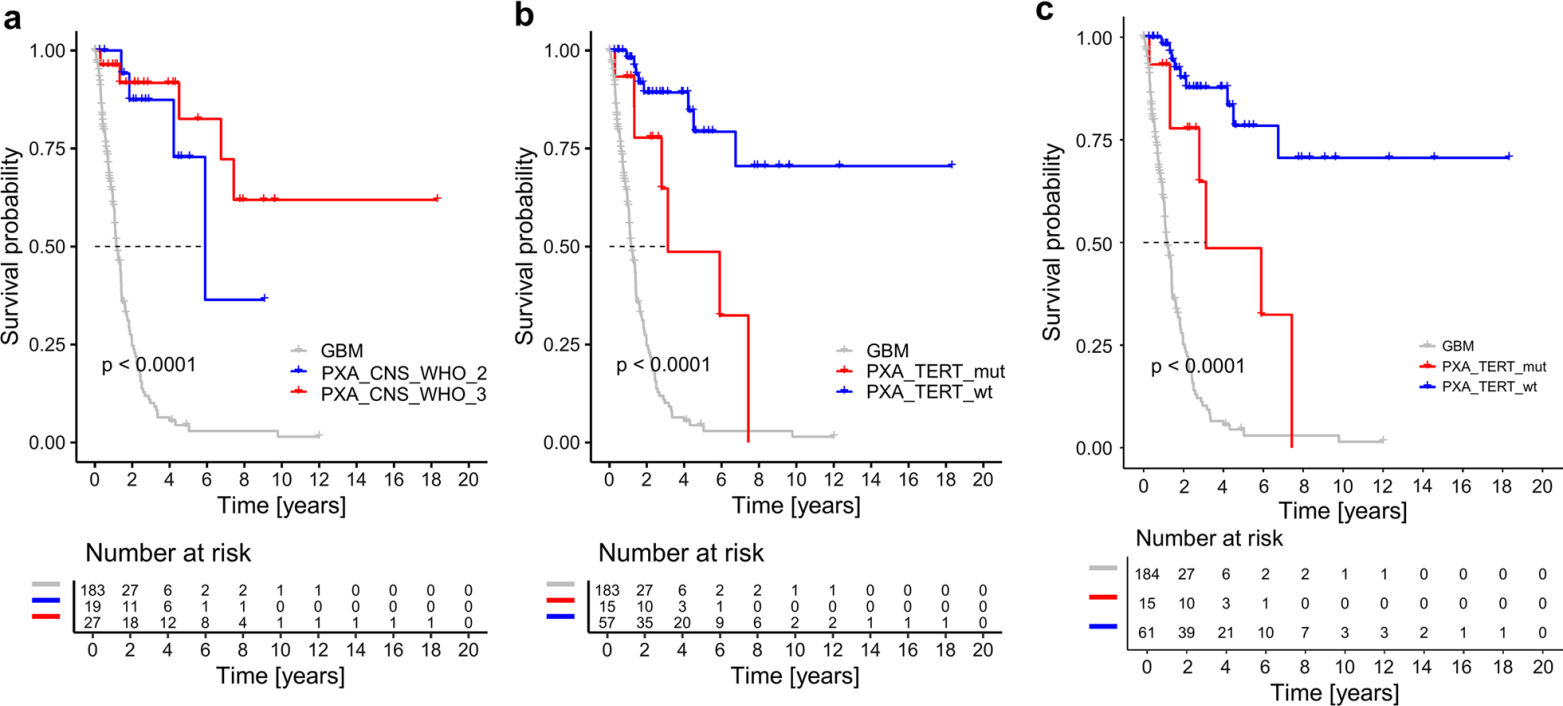
Overall survival (Kaplan–Meier curve) of patients in cohort mcPXA stratified after CNS WHO grade (**a**) and *pTERT* status (**b**, **c**). (**b**) includes tumors classified via the brain tumor classifier and (**c**) includes tumors classified via tSNE. *pTERT* mutant mcPXA revealed significantly worse overall survival compared to wild type mcPXA (log rank P* < 0.0001). CNS WHO grade did not show any prognostic significance in mcPXAs (log rank P > 0.05)

Kaplan–Meier analysis of survival data did not reveal a significant difference between tumors of mcPXA that would be considered as “grade 2” and “grade 3”, (Fig. 3a).

### Canonical pTERT mutations associate with worse prognosis in mcPXA

Kaplan–Meier analysis of 72 mcPXA tumors stratified for *pTERT* status revealed a significant association between canonical *pTERT* mutations and clinical outcome (Fig. 3b). Patients with *pTERT*-mutant mcPXA had a worse prognosis compared to patients with *pTERT*-wildtype mcPXA tumors (Fig. 3b, n_mut_ = 15, n_wt_ = 57, median OS not reached; log rank p < 0.001). Combining mcPXA with those unclassifiable tumors from histPXA cohort that were attributed to the molecular cluster PXA by tSNE, showed the same effect of *pTERT* mutation on survival (Fig. 3c). There was no significant difference in frequency of *pTERT* mutations between “grade 2” and “grade 3” mcPXAs. McGBM fared worse than both *pTERT*-wildtype and *pTERT*-mutant mcPXA (Fig. 3b, c).

### BRAF V600E mutations in mcPXA

Similarly, 77 mcPXA tumors with available *BRAF* status and survival data were analyzed via Kaplan–Meier analysis to evaluate the effect of *BRAF V600E* mutation on clinical outcome. The presence of *BRAF V600E* mutation in mcPXA was associated with a slightly better prognosis (Additional file 5: Fig. 2b, n_mut_ = 65, n_wt_ = 12; p = 0.04).

### mcPXA with histological diagnosis GBM fares worse than mcPXA with initial histological diagnosis PXA

McPXA patients with histological diagnosis GBM (n = 29) showed shorter survival compared to mcPXA patients with definite histological diagnosis PXA (n = 26) or differential diagnosis PXA (n = 3) (Additional file 5: Fig. 2a, median OS not reached, p = 0.013). Seven mcPXA tumors with initial histological diagnosis GBM (4/29) and epithelioid GBM (3/29) showed in addition + 7/-10 signature. Of these, one tumor had combination of *pTERT* and *BRAF V600E* mutations and was initially diagnosed as epithelioid GBM. One tumor with initial histological diagnosis GBM had *BRAF V600E* mutation and the rest were *pTERT-* and *BRAF V600E-*wildtype. None of those exhibited *EGFR* amplification. *pTERT* mutation was a more frequent event in tumors with histological diagnosis GBM assigned to mcPXA (n = 12/32 mcPXA-histGBM) compared to tumors with histological diagnosis PXA assigned to mcPXA (n = 3/35 mcPXA-histPXA). *BRAF V600E* was a frequent mutation in both groups (n = 31/39 mcPXA-histGBM, n = 52/57 mcPXA-histPXA) and did not show a significant difference (Additional file 2: Table 2).

### mcPXA shows a distinct copy number profile from mcGBM

McPXA showed a distinct copy number profile compared to that of mcGBM as previously described (Additional file 5: Fig. 3) [2, 3, 22]. Loss of chromosome 9p (n = 114 of 220; 52%) and homozygous deletion of *CDKN2A/B* (n = 196 of 220; 89%) were the most common CNVs in PXA, compatible with our previous publication on DNA methylation-based classification of tumors [3]. None of the copy number variations including alterations on specific gene locations with relevance for astrocytic gliomas as well as broader copy number gains and losses, had a significant effect on prognosis in mcPXA (n = 77; log rank P > 0.05). We did not find any differences in DNA methylation pattern of gene sets in CNS WHO grade 2 tumors compared to grade 3 mcPXAs (Additional file 5: Fig. 4). Thirteen mcPXAs had a + 7/−10 signature in combination with *CDKN2A/B* deletion; however, none of them had amplifications typical for GBM such as *CDK4, CDK6, EGFR, MDM2, MDM4* and *MET*. Of note, the 13 cases with + 7/−10 signature had a high number of other copy number variations indicating an increased genomic instability (Additional file 2: Table 2). All of these cases had a calibrated score 0.9 or higher for mcPXA. Survival data were available only for 5 of these cases precluding further analysis (Additional file 2: Table 2).

## Discussion

### Composition of cohorts histPXA and mcPXA

PXAs are challenging tumors for diagnosis. Distinction from glioblastoma variants, most importantly epithelioid glioblastoma, and glioneuronal tumors, specifically ganglioglioma, is a common diagnostic problem. In our histPXA cohort of 144 tumors, 79 tumors received a clear classifier output. 62 of these belonged to mcPXA (79%) while 17 lesions were allotted to other methylation classes (Additional file 5: Fig. 5). A previous study with a smaller cohort size also showed a majority of histologically diagnosed PXA (87%, n = 40 of 46) fall into the methylation class PXA [26]. Similar to our study, the cases in this study classified to the methylation classes of PXA (n = 40), ganglioglioma or pilocytic astrocytoma (n = 2), anaplastic pilocytic astrocytoma (n = 2) and control tissues (n = 2). However, the size of histPXA cohort examined for methylation class (n = 46) in this study is significantly smaller than our study, that might explain the higher variability among methylation class assignments in our histPXA cohort (n = 144). Our observation of 65 tumors in cohort histPXA, not receiving a brain tumor classifier output for a distinct methylation class above threshold, was surprising. This contradicts our experience from our extensive validation set of 1155 tumors for which a classifier output above threshold was achieved in 977 tumors (85%) [2]. A low quality tissue could not describe the low performance in the methylation analysis. According to our purity index analyses, the purity and consequently the DNA quality was the same in classifiable and non-classifiable cases. One reason for subthreshold classifier output could be non-resolvable tumors meaning that for a specific tumor sample no corresponding methylation class has been included in the classifier so far. The typical other main reason for subthreshold output could be low tumor cell fraction in the sample. Samples with the highest calibrated score for control-inflammation are examples of such cases. In those cases, the inflammatory component is very prominent. Similarly, biopsies from the tumor periphery might fall in the methylation class control-reactive. Of 65 tumors without threshold reaching classifier output 38 were allotted to the methylation classes PXA, GBM or control tissue in tSNE analysis. This suggests the presence of a high fraction of non-neoplastic brain tissue in these cases and hints towards a tendency to favor the diagnosis of PXA in the infiltration zone of glial tumors.

McPXA encompassing 220 tumors, 73 diagnosed as PXA, 50 as GBM, 50 as glioma, 31 as glioneuronal tumors and 16 receiving an “other” diagnosis. We attribute this surprisingly high variation to a wider than currently recognized morphological spectrum encountered in PXA. McPXA has only been formed on the basis of DNA methylation analysis and includes those 73 patients from cohort histPXA receiving mcPXA output (Additional file 2: Table 2). Noteworthy 50 (23%) of the tumors in cohort mcPXA have been diagnosed as GBM, with 9 being diagnosed as epithelioid GBM. Interestingly, only 7/50 of these lesions carried a 7/10 signature while none exhibited *EGFR* amplification. The separation of the variant of epithelioid glioblastoma from PXA poses special problems. This is for the overlapping morphology with PXA as well as for the frequent occurrence of *BRAF V600E* mutations in epithelioid GBM, another feature shared with PXA. Recent studies challenge the existence of epithelioid GBM [1, 6, 13]. The latter study addressed both, pediatric and adult epithelioid GBM by DNA methylation analysis and reported an evident heterogeneity in this group. Tumors could clearly be allotted to methylation profiles from well-established tumor entities including PXA and RTK I GBM for younger patients and IDHwt GBM for older patients. Given the overlapping morphological and molecular features, it is not unexpected that tumors diagnosed as GBM constitute the second numerous fraction in mcPXA. Another 31 tumors have been diagnosed as either gangliogliomas or other glioneuronal tumors. This is in line with common experience of GBM and ganglioglioma constituting the most relevant differential diagnoses at the high- and low-grade ends of the morphological PXA spectrum. We conclude from the comparison of DNA methylation- and histology-based classification in cohort mcPXA that the morphological spectrum of PXA might be wider than previously assumed.

## Parameters associated with prognosis in histPXA and mcPXA

Prognosis associated parameters are eminent for tumor grading. Grading of PXA relies on mitotic activity. Five or more mitoses per 10 high-power fields render a PXA as CNS WHO grade 3 [8, 14]. Employing these WHO criteria to the cohort histPXA demonstrates separation of PXA in two groups of different survival with patients exhibiting a high mitotic count faring worse, however with a p-value slightly higher than statistically significant, mainly due to the small sample size (Fig. 2). In this regard, our findings were in accordance with previous studies, rendering the survival significance of mitotic activity in PXA [26]. In contrast, employing WHO grading criteria to tumors in cohort mcPXA fails to separate this group into groups of distinct survival (Fig. 3a). This discrepancy can be explained: histPXA cohort includes tumors that in methylation classification qualifying for GBM on one end of the malignancy spectrum and ganglioglioma on the other end. The presence of GBM typically associated with increased proliferation and a very unfavorable prognosis, as well as the presence of ganglioglioma with low proliferation and a very good prognosis, strongly augments the impact of mitosis in survival analysis. Thus, the prognostic role of mitoses in WHO grading of PXA may be rooted in the examination of heterogeneous tumor cohorts with inclusion of unrecognized highly malignant and very benign tumors. In fact, this very much parallels the problems with grading of diffuse astrocytoma. There, the presence of mitoses has lost much impact in grading, after IDH status was implemented, in order to remove underdiagnosed GBM from study cohorts [22, 28]. The failure of WHO criteria to separate tumors with the methylation profile of PXA can be explained by the epigenetically homogenous nature of cohort mcPXA. One might argue that in Vaubel et. al. study [26], most of the histPXAs were classified to mcPXA and mitosis was still a significant prognostic factor. Likewise, a majority of our histPXA were classified to mcPXA and we found mitotic count a relevant prognostic factor in histPXAs. However, in Vaubel et. al study, a reverse approach examining a cohort of mcPXA with histological variability was missing and our results concerning the prognostic significance of mitotic activity in mcPXA cohort cannot be compared to that study.

With classical WHO criteria not successful in stratifying for survival in mcPXA, we searched for other parameters potentially useful as discriminators between tumor grades. The morphological parameters, cellularity, Ki67-index, presence/absence of necrosis and presence/absence of microvascular proliferation did not stratify for survival. Neither did *CDKN2A/2B* status, being very frequent in PXA. The single most relevant parameter for distinguishing cohort mcPXA patients with favorable from those with poor outcome was *pTERT* status. Patients with one of the two canonical mutations in the promoter of the telomerase reverse transcriptase (*TERT*) gene fared significantly worse than patients with a *pTERT* wild type status (Fig. 3b, c). Thus, in a DNA methylation-based defined PXA cohort, *pTERT* mutation appears to be a very strong grading parameter. In line with our data, two recent studies in PXA have seen *pTERT* mutations in progressed PXA [7, 20]. Because *pTERT* mutations in PXA are less common than the count of 5 mitoses or more per 10 high-power fields, this may affect the frequency of anaplastic PXA diagnosis. In our cohort, mcPXA data were available from 72 patients with *pTERT* mutation detected in 15 (21%). Another study restricted to anaplastic PXA detected 5 *pTERT* mutations in 15 tumors (33%) [20]. In our mcPXA cohort, patients having received the histological diagnosis of GBM fared worse (Additional file 5: Fig. 2a). This prompted us to question our observation of clear morphological parameters lacking for grading mcPXA. It turned out that *pTERT* mutant tumors predominantly received the morphological diagnosis of GBM (n = 12 of 17), and that survival of mcPXA was dependent on *pTERT* status (Fig. 3).

In our series, DNA methylation profiles did not emerge as grading parameter separating PXA with favorable from those with poor survival. However, a previous study has reported a higher degree of DNA methylation in 2016 WHO grade III PXA compared to WHO grade II counterparts [16]. *BRAF V600E* mutation occurred in 117/220 mcPXA and was associated with better survival (65 of77 with survival data, p = 0.04). This association has been reported previously. Some studies have shown an association of this mutation with better prognosis [8, 23]. However, *BRAF V600E* mutation and further MAPK activating molecular alterations are frequent events in low grade glial and glioneuronal tumors [5, 21, 23, 27]. The prognostic impact of *BRAF* mutation is still a matter of debate and requires further studies on frequency of all *MAPK* activating alterations in PXAs.

## Conclusion

This study demonstrates high methylation class heterogeneity within the tumors with histological diagnosis PXA. Diagnostic evaluation of tumors within the morphological scope of PXA can be assisted by DNA methylation array analysis. Our data suggest canonical *pTERT* mutations as robust indicators for poor prognosis in mcPXA.

## Supplementary Information

**Additional file 1.** List of tumors in cohort histPXA including 144 tumors.

**Additional file 2.** List of tumors in cohort mcPXA including 220 tumors.

**Additional file 3.** Composition of cohorts mcPXA and histPXA in numbers.

**Additional file 4.** tSNE plot of histPXA cases with the set of reference samples underlying the classifier version v11b4; “histPXA a” represents cases with a calibrated score less than 0.9 in v11b4 classifier, “histPXA b” represents cases with a calibrated score of 0.9 or higher in v11b4 classifier.

**Additional file 5.** Histological features observed in pleomorphic xanthoastrocytomas, (a) classical giant pleomorphic cells with multiple nuclei and prominent nucleolus, (b) extensive perivascular inflammatory infiltrates, (c) endothelial proliferations, (d) extensive myxoid matrix, (e) pseudopalisading necrosis, (f) pseudopapillary growth pattern, (g) biphasic pattern with spindle cell component and giant pleomorphic cell component, (h) extensive calcification, (i) small round blue cell morphology, (j) a thrombosed vessel; all depicted tumors had a maximum calibrated score above 0.9 for mcPXA; Supplementary figure 2 Overall survival (Kaplan-Meier curve) of patients in cohort mcPXA stratified after initial histological diagnosis (a) and BRAF V600E status (b); Supplementary figure 3 Typical copy number profile of mcPXA (upper panel) compared to that of mcGBM (lower panel); Supplementary figure 4 Copy number summary of cohort mcPXA altogether and stratified after WHO grade; Supplementary figure 5: The composition of cohorts histPXA and mcPXA, (a) 220 mcPXA cases with their histological composition, (b) 144 histPXA cases with their methylation class assignments (v11b4).

**Additional file 6.** List of abbreviations.

## Data Availability

Due to the patients’ confidentiality, the raw idat data of the patients generated via DNA methylation array analysis cannot be shared. The rest of the data generated or analyzed during this study, are included in this published article and its supplementary information files.
