# Supplementary material for: Pleomorphic xanthoastrocytoma is a heterogeneous entity with pTERT mutations prognosticating shorter survival
Source: Acta Neuropathol Commun. 2022 Jan 10;10:5. doi: 10.1186/s40478-021-01308-1 (PMC8751269; doi:10.1186/s40478-021-01308-1)
Supplement: Supplementary file 3 — Additional file 3. Composition of cohorts mcPXA and histPXA in numbers. [file 40478_2021_1308_MOESM3_ESM.pdf]

Supplementary table 3: Composition of cohorts mcPXA and histPXA in numbers

| Cohort | Number of cases (n) |
|---|---|
| **mcPXA** | 220 |
| • **Availabe survival data** | 77 |
| • **pTERT sequencing** | 95 |
| • **BRAF sequencing** | 143 |
| • **Histological reevaluation in HD** | 46 |
| **histPXA** | 144 |
| • **Availabe survival data** | 53 |
| • **pTERT sequencing** | 50 |
| • **BRAF sequencing** | 73 |
| • **Histological reevaluation in HD** | 19 |
