# Supplementary figures and images for: Pleomorphic xanthoastrocytoma is a heterogeneous entity with pTERT mutations prognosticating shorter survival
Source: Acta Neuropathol Commun. 2022 Jan 10;10:5. doi: 10.1186/s40478-021-01308-1 (PMC8751269; doi:10.1186/s40478-021-01308-1)

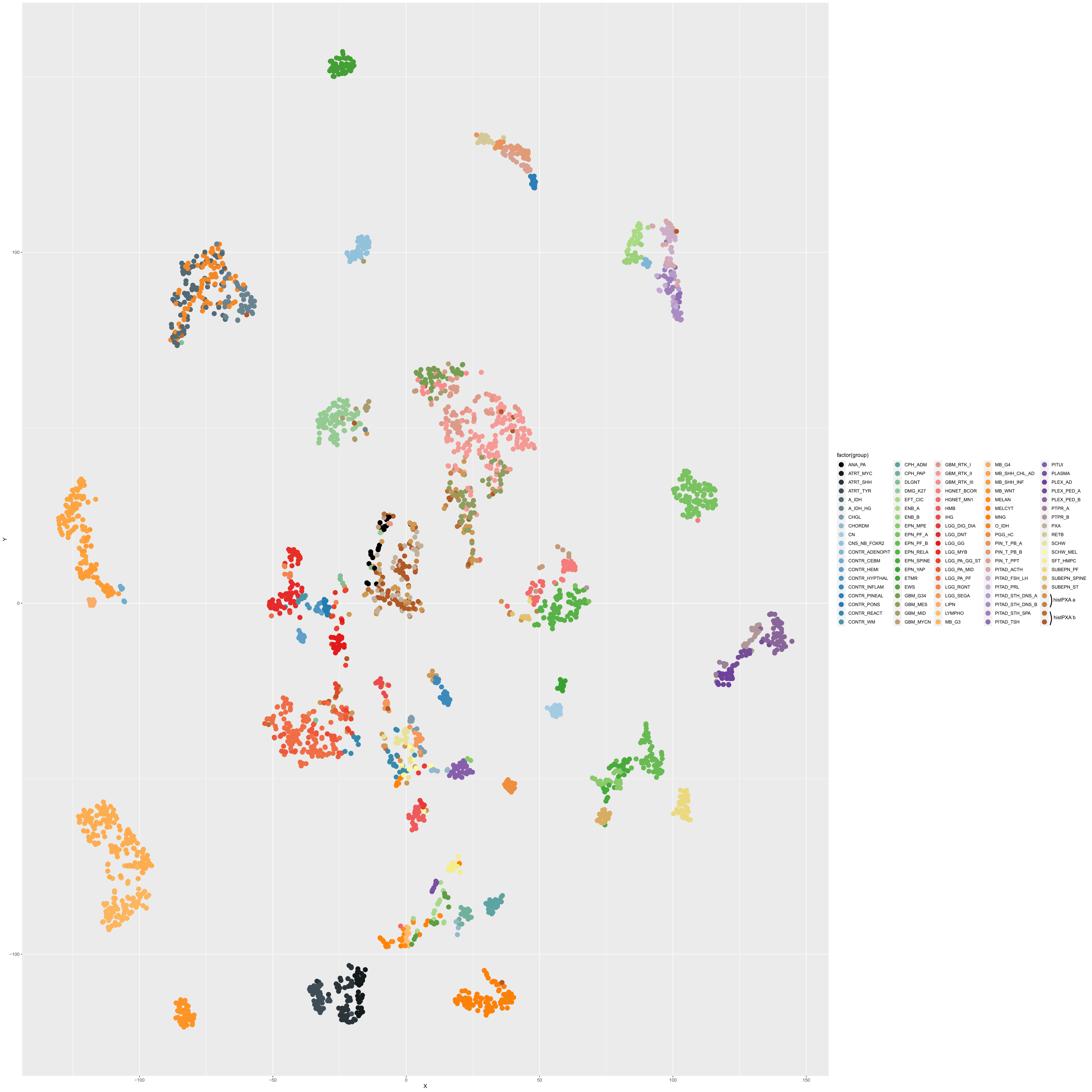

Supplement: Supplementary file 4 — Additional file 4. tSNE plot of histPXA cases with the set of reference samples underlying the classifier version v11b4; “histPXA a” represents cases with a calibrated score less than 0.9 in v11b4 classifier, “histPXA b” represents cases with a calibrated score of 0.9 or higher in v11b4 classifier. [file 40478_2021_1308_MOESM4_ESM.pdf]
