# Supplementary material for: Pleomorphic xanthoastrocytoma is a heterogeneous entity with pTERT mutations prognosticating shorter survival
Source: Acta Neuropathol Commun. 2022 Jan 10;10:5. doi: 10.1186/s40478-021-01308-1 (PMC8751269; doi:10.1186/s40478-021-01308-1)
Supplement: Supplementary file 5 — Additional file 5. Histological features observed in pleomorphic xanthoastrocytomas, (a) classical giant pleomorphic cells with multiple nuclei and prominent nucleolus, (b) extensive perivascular inflammatory infiltrates, (c) endothelial proliferations, (d) extensive myxoid matrix, (e) pseudopalisading necrosis, (f) pseudopapillary growth pattern, (g) biphasic pattern with spindle cell component and giant pleomorphic cell component, (h) extensive calcification, (i) small round blue cell morphology, (j) a thrombosed vessel; all depicted tumors had a maximum calibrated score above 0.9 for mcPXA; Supplementary figure 2 Overall survival (Kaplan-Meier curve) of patients in cohort mcPXA stratified after initial histological diagnosis (a) and BRAF V600E status (b); Supplementary figure 3 Typical copy number profile of mcPXA (upper panel) compared to that of mcGBM (lower panel); Supplementary figure 4 Copy number summary of cohort mcPXA altogether and stratified after WHO grade; Supplementary figure 5: The composition of cohorts histPXA and mcPXA, (a) 220 mcPXA cases with their histological composition, (b) 144 histPXA cases with their methylation class assignments (v11b4). [file 40478_2021_1308_MOESM5_ESM.pdf]

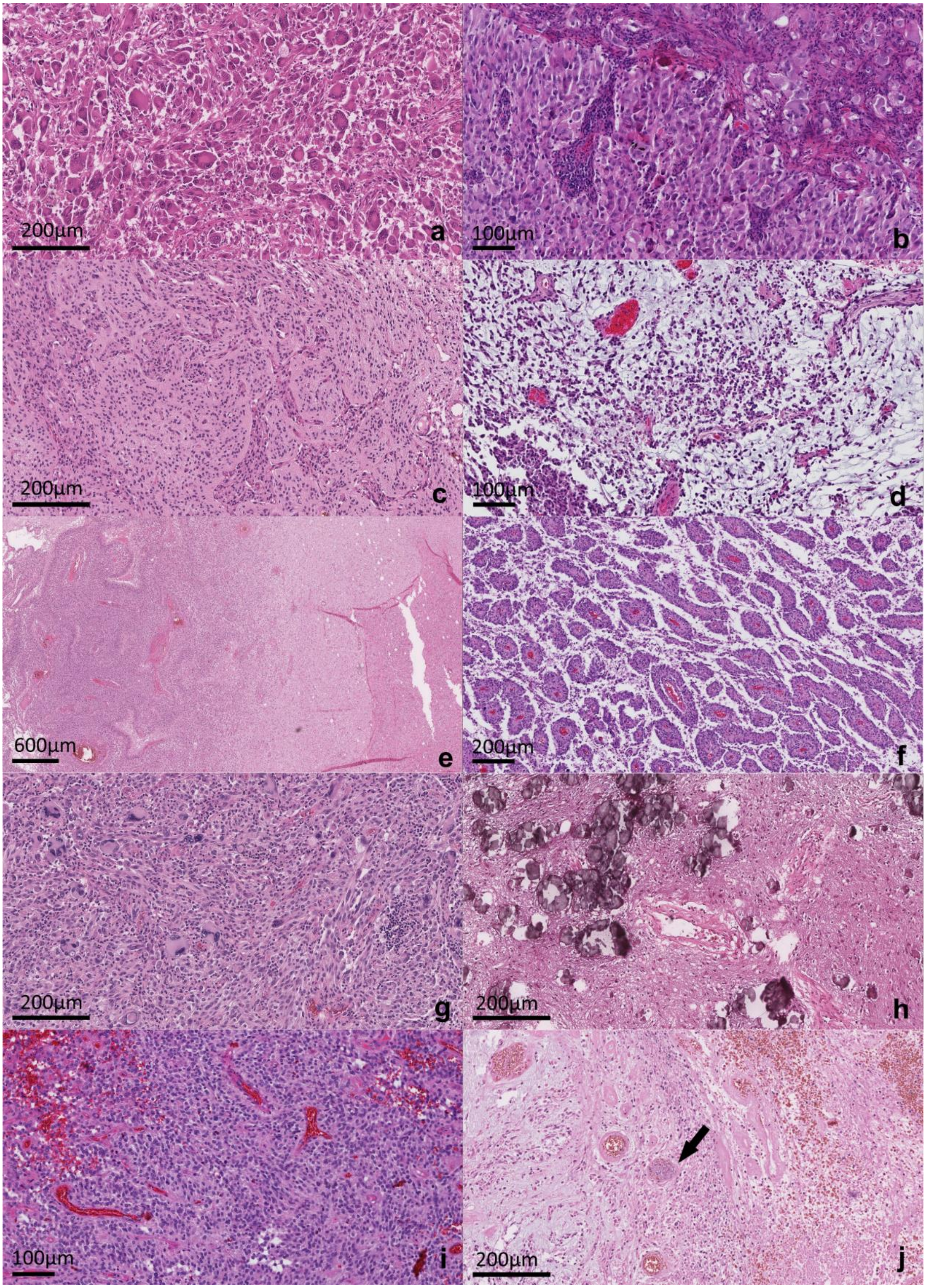

Supplementary figure 1 Histological features observed in pleomorphic xanthoastrocytomas, **(a)** classical giant pleomorphic cells with multiple nuclei and prominent nucleolus, **(b)** extensive perivascular inflammatory infiltrates, **(c)** endothelial proliferations, **(d)** extensive myxoid matrix, **(e)** pseudopalisading necrosis, **(f)** pseudopapillary growth pattern, **(g)** biphasic pattern with spindle cell component and giant pleomorphic cell component, **(h)** extensive calcification, **(i)** small round blue cell morphology, **(j)** a thrombosed vessel; all depicted tumors had a maximum calibrated score above 0.9 for mcPXA

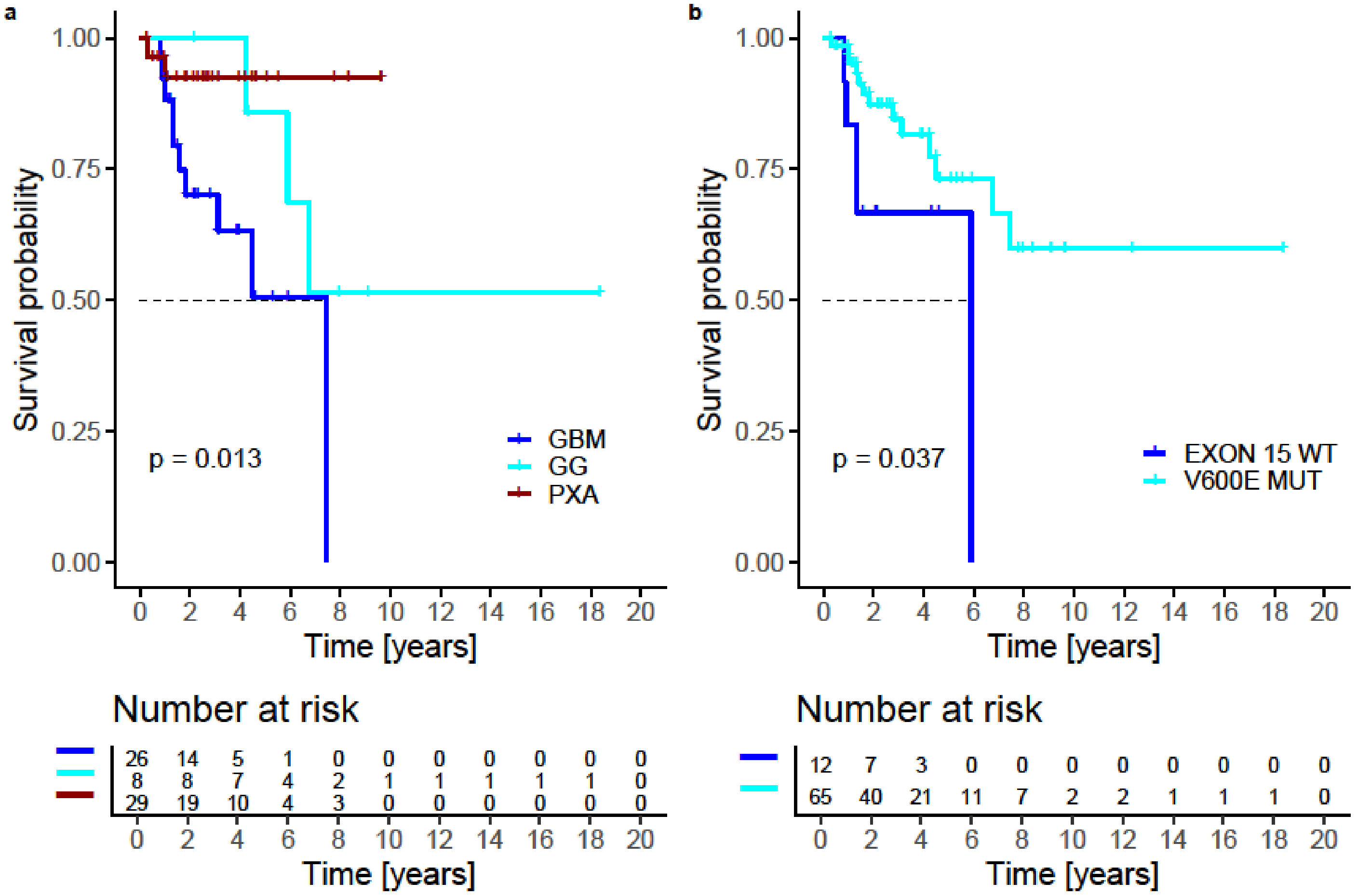

Supplementary figure 2 Overall survival (Kaplan-Meier curve) of patients in cohort mcPXA stratified after initial histological diagnosis (a) and BRAF V600E status (b)

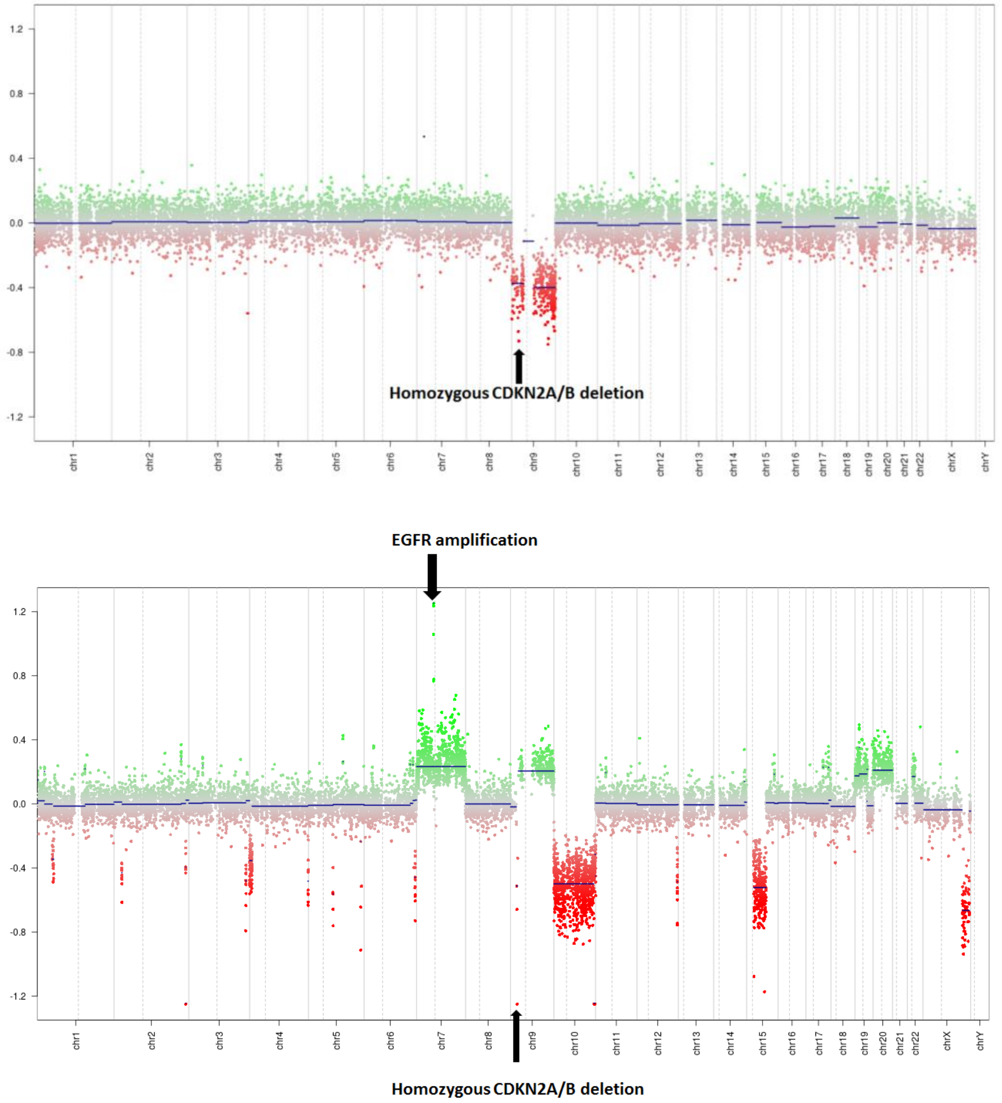

Supplementary figure 3 Typical copy number profile of mcPXA (upper panel) compared to that of mcGBM (lower panel)

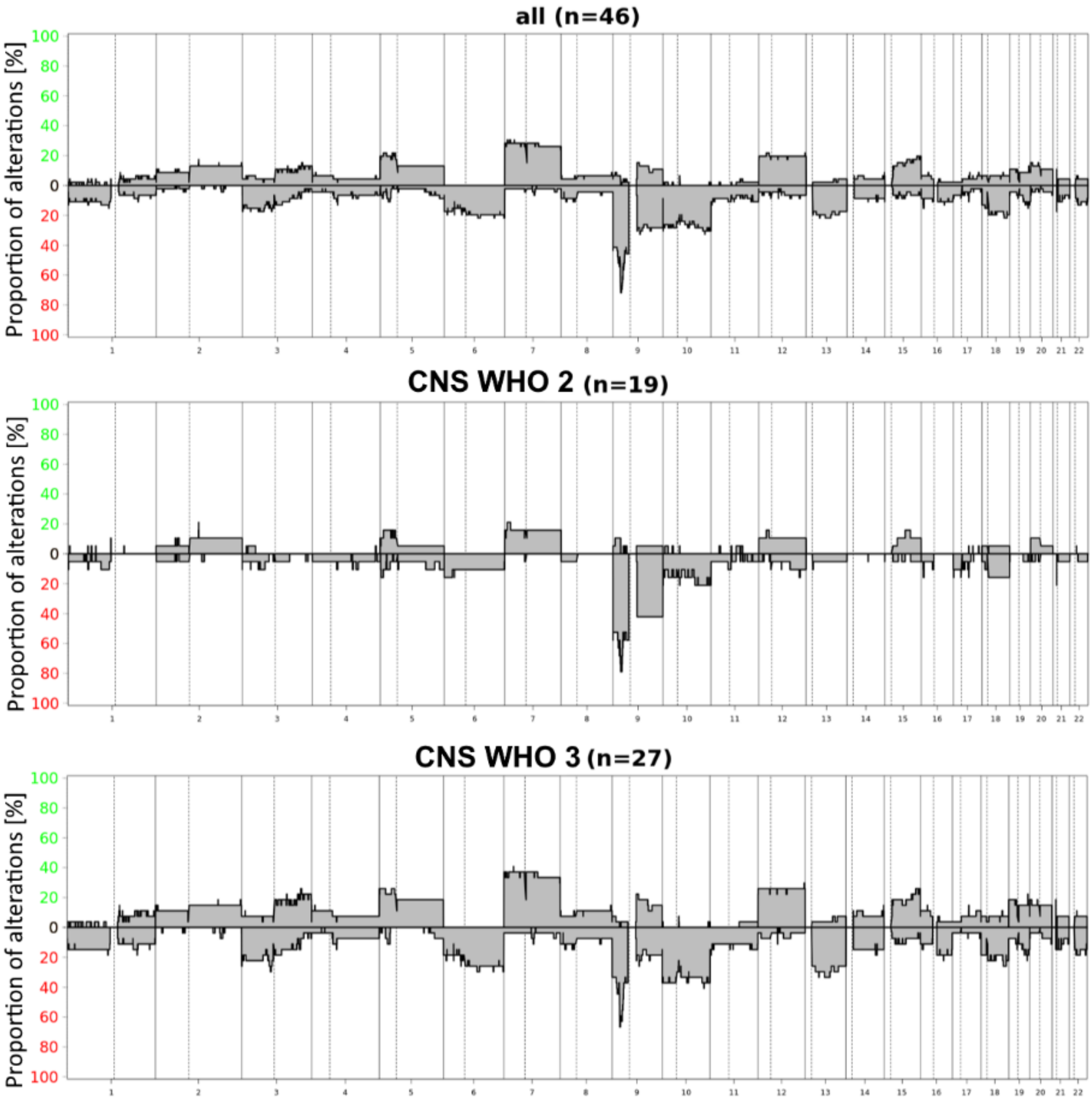

Supplementary figure 4 Copy number summary of cohort mcPXA altogether and stratified after CNS WHO grade

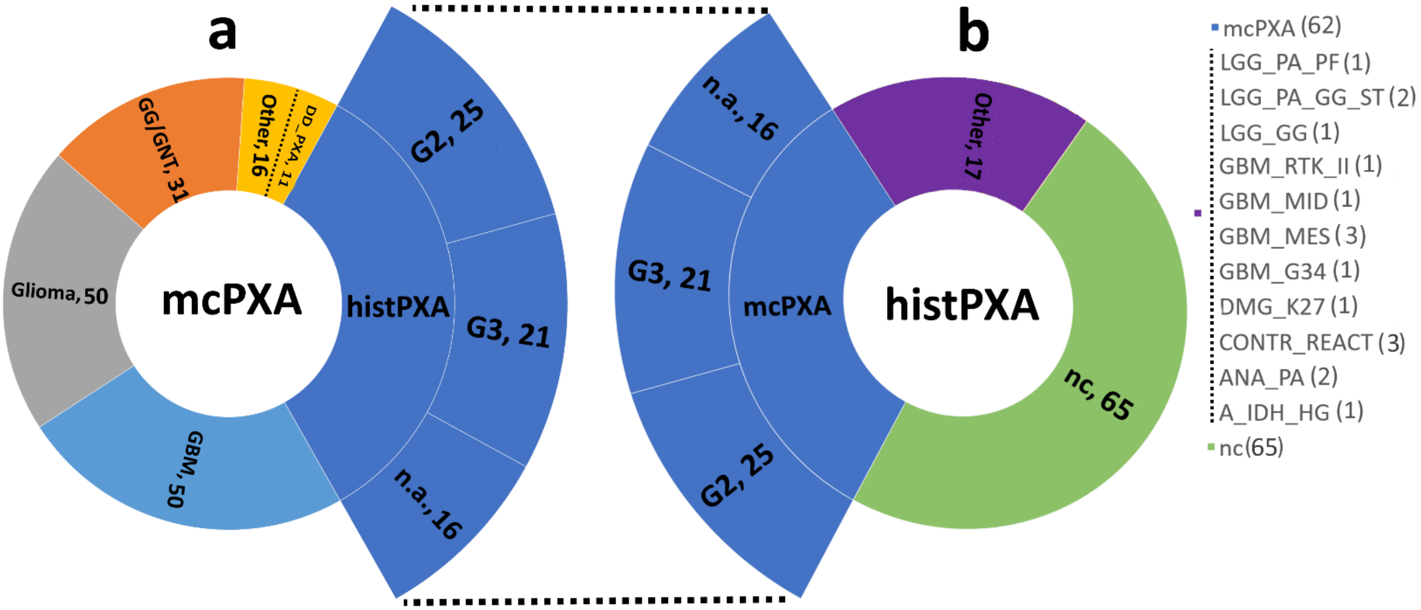

Supplementary figure 5: The composition of cohorts histPXA and mcPXA, (a) 220 mcPXA cases with their histological composition, (b) 144 histPXA cases with their DNA methylation class assignments (v11b4)
