# Supplementary material for: Pleomorphic xanthoastrocytoma is a heterogeneous entity with pTERT mutations prognosticating shorter survival
Source: Acta Neuropathol Commun. 2022 Jan 10;10:5. doi: 10.1186/s40478-021-01308-1 (PMC8751269; doi:10.1186/s40478-021-01308-1)
Supplement: Supplementary file 6 — Additional file 6. List of abbreviations. [file 40478_2021_1308_MOESM6_ESM.pdf]

List of abbreviations	
histPXA	histological pleomorphic xanthoastrocytoma (cohort)
mcPXA	methylation class pleomorphic xanthoastrocytoma (cohort)
E-GBM	epithelioid glioblastoma
GBM	glioblastoma
GG	ganglioglioma
GNT	glioneuronal tumors
AT/RT	atypical teratoid rhabdoid tumor
FCD	focal cortical dysplasia
n.a.	infromation not available
nc	not classifiable
PXA	pleomorphic xanthoastrocytoma
DD_PXA	differential diagnosis pleomorphic xanthoastrocytoma
A IDH	methylation class IDH glioma, subclass astrocytoma
A IDH, HG	methylation class IDH glioma, subclass high grade astrocytoma
ANA PA	methylation class anaplastic pilocytic astrocytoma
ATRT, MYC	methylation class atypical teratoid/rhabdoid tumor, subclass MYC
ATRT, SHH	methylation class atypical teratoid/rhabdoid tumor, subclass SHH
ATRT, TYR	methylation class atypical teratoid/rhabdoid tumor, subclass TYR
CHGL	methylation class chordoid glioma of the third ventricle
CHORDM	methylation class chordoma
CN	methylation class central neurocytoma
CNS NB, FOXR2	methylation class CNS neuroblastoma with FOXR2 activation
CONTR, ADENOPIT	methylation class control tissue, pituitary gland anterior lobe
CONTR, CEBM	methylation class control tissue, cerebellar hemisphere
CONTR, HEMI	methylation class control tissue, hemispheric cortex
CONTR, HYPTHAL	methylation class control tissue, hypothalamus
CONTR, INFLAM	methylation class control tissue, inflammatory tumor microenvironment
CONTR, PINEAL	methylation class control tissue, pineal gland
CONTR, PONS	methylation class control tissue, pons
CONTR, REACT	methylation class control tissue, reactive tumor microenvironment
CONTR, WM	methylation class control tissue, white matter
CPH, ADM	methylation class craniopharyngioma, adamantinomatous
CPH, PAP	methylation class craniopharyngioma, papillary
DLGNT	methylation class diffuse leptomeningeal glioneuronal tumor
DMG, K27	methylation class diffuse midline glioma H3 K27M mutant
EFT, CIC	methylation class CNS Ewing sarcoma family tumor with CIC alteration
ENB, A	methylation class esthesioneuroblastoma, subclass A
ENB, B	methylation class esthesioneuroblastoma, subclass B
EPN, MPE	methylation class ependymoma, myxopapillary
EPN, PF A	methylation class ependymoma, posterior fossa group A
EPN, PF B	methylation class ependymoma, posterior fossa group B
EPN, RELA	methylation class ependymoma, RELA fusion
EPN, SPINE	methylation class ependymoma, spinal
EPN, YAP	methylation class ependymoma, YAP fusion
ETMR	methylation class embryonal tumor with multilayered rosettes
EWS	methylation class Ewing sarcoma
GBM, G34	methylation class glioblastoma, IDH wildtype, H3.3 G34 mutant
GBM, MES	methylation class glioblastoma, IDH wildtype, subclass mesenchymal
GBM, MID	methylation class glioblastoma, IDH wildtype, subclass midline
GBM, MYCN	methylation class glioblastoma, IDH wildtype, subclass MYCN

GBM, RTK I	methylation class glioblastoma, IDH wildtype, subclass RTK I
GBM, RTK II	methylation class glioblastoma, IDH wildtype, subclass RTK II
GBM, RTK III	methylation class glioblastoma, IDH wildtype, subclass RTK III
HGNET, BCOR	methylation class CNS high grade neuroepithelial tumor with BCOR alteration
HGNET, MN1	methylation class CNS high grade neuroepithelial tumor with MN1 alteration
HMB	methylation class hemangioblastoma
IHG	methylation class infantile hemispheric glioma
LGG, DIG/DIA	methylation class low grade glioma, desmoplastic infantile astrocytoma / ganglioglioma
LGG, DNT	methylation class low grade glioma, dysembryoplastic neuroepithelial tumor
LGG, GG	methylation class low grade glioma, ganglioglioma
LGG, MYB	methylation class low grade glioma, MYB/MYBL1
LGG, PA MID	methylation class low grade glioma, subclass midline pilocytic astrocytoma
LGG, PA PF	methylation class low grade glioma, subclass posterior fossa pilocytic astrocytoma
LGG, PA/GG ST	methylation class low grade glioma, subclass hemispheric pilocytic astrocytoma and ganglioglioma
LGG, RGNT	methylation class low grade glioma, rosette forming glioneuronal tumor
LGG, SEGA	methylation class low grade glioma, subependymal giant cell astrocytoma
LIPN	methylation class cerebellar liponeurocytoma
LYMPHO	methylation class lymphoma
MB, G3	methylation class medulloblastoma, subclass group 3
MB, G4	methylation class medulloblastoma, subclass group 4
MB, SHH CHL AD	methylation class medulloblastoma, subclass SHH A (children and adult)
MB, SHH INF	methylation class medulloblastoma, subclass SHH B (infant)
MB, WNT	methylation class medulloblastoma, WNT
MELAN	methylation class melanoma
MELCYT	methylation class melanocytoma
MNG	methylation class meningioma
O IDH	methylation class IDH glioma, subclass 1p/19q codeleted oligodendroglioma
PGG, nC	methylation class paraganglioma, spinal non-CIMP
PIN T, PB A	methylation class pineoblastoma group A / intracranial retinoblastoma
PIN T, PB B	methylation class pineoblastoma group B
PIN T, PPT	methylation class pineal parenchymal tumor
PITAD, ACTH	methylation class pituitary adenoma, ACTH
PITAD, FSH LH	methylation class pituitary adenoma, FSH/LH
PITAD, PRL	methylation class pituitary adenoma, prolactin
PITAD, STH DNS A	methylation class pituitary adenoma, STH densely granulated, group A
PITAD, STH DNS B	methylation class pituitary adenoma, STH densely granulated, group B
PITAD, STH SPA	methylation class pituitary adenoma, STH sparsely granulated
PITAD, TSH	methylation class pituitary adenoma, TSH
PITUI, SCO, GCT	methylation class pituicytoma / granular cell tumor / spindle cell oncocytoma
PLASMA	methylation class plasmacytoma
PLEX, AD	methylation class plexus tumor, subclass adult
PLEX, PED A	methylation class plexus tumor, subclass paediatric A
PLEX, PED B	methylation class plexus tumor, subclass paediatric B
PTPR, A	methylation class papillary tumor of the pineal region group A
PTPR, B	methylation class papillary tumor of the pineal region group B
PXA (tSNE reference class)	methylation class (anaplastic) pleomorphic xanthoastrocytoma

RETB	methylation class retinoblastoma
SCHW	methylation class schwannoma
SCHW, MEL	methylation class melanotic schwannoma
SFT HMPC	methylation class solitary fibrous tumor / hemangiopericytoma
SUBEPN, PF	methylation class subependymoma, posterior fossa
SUBEPN, SPINE	methylation class subependymoma, spinal
SUBEPN, ST	methylation class subependymoma, supratentorial
